# Sensory-motor deficits and neurofilament disorganization in gigaxonin-null mice

**DOI:** 10.1186/1750-1326-6-25

**Published:** 2011-04-12

**Authors:** Thibault Ganay, Alexia Boizot, Renaud Burrer, Jean Paul Chauvin, Pascale Bomont

**Affiliations:** 1 Inserm Unité 901, Marseille, 13009, France; 2 Université de la Méditerranée, UMR S901 Aix-Marseille 2, 13009, France; 3 INMED, Marseille 13009, France; 4 Ibdml, UMR6216, Université de la Méditerranée, 13009, France; 5 DIMNP, Université Montpellier II, CNRS (UMR 5235), Montpellier, France

## Abstract

**Background:**

Giant Axonal Neuropathy (GAN) is a fatal neurodegenerative disorder with early onset characterized by a severe deterioration of the peripheral and central nervous system, involving both the motor and the sensory tracts and leading to ataxia, speech defect and intellectual disabilities. The broad deterioration of the nervous system is accompanied by a generalized disorganization of the intermediate filaments, including neurofilaments in neurons, but the implication of this defect in disease onset or progression remains unknown. The identification of gigaxonin, the substrate adaptor of an E3 ubiquitin ligase, as the defective protein in GAN allows us to now investigate the crucial role of the gigaxonin-E3 ligase in sustaining neuronal and intermediate filament integrity. To study the mechanisms controlled by gigaxonin in these processes and to provide a relevant model to test the therapeutic approaches under development for GAN, we generated a Gigaxonin-null mouse by gene targeting.

**Results:**

We investigated for the first time in Gigaxonin-null mice the deterioration of the motor and sensory functions over time as well as the spatial disorganization of neurofilaments. We showed that gigaxonin depletion in mice induces mild but persistent motor deficits starting at 60 weeks of age in the 129/SvJ-genetic background, while sensory deficits were demonstrated in C57BL/6 animals. In our hands, another gigaxonin-null mouse did not display the early and severe motor deficits reported previously. No apparent neurodegeneration was observed in our knock-out mice, but dysregulation of neurofilaments in proximal and distal axons was massive. Indeed, neurofilaments were not only more abundant but they also showed the abnormal increase in diameter and misorientation that are characteristics of the human pathology.

**Conclusions:**

Together, our results show that gigaxonin depletion in mice induces mild motor and sensory deficits but recapitulates the severe neurofilament dysregulation seen in patients. Our model will allow investigation of the role of the gigaxonin-E3 ligase in organizing neurofilaments and may prove useful in understanding the pathological processes engaged in other neurodegenerative disorders characterized by accumulation of neurofilaments and dysfunction of the Ubiquitin Proteasome System, such as Amyotrophic Lateral Sclerosis, Huntington's, Alzheimer's and Parkinson's diseases.

## Background

Giant Axonal Neuropathy (GAN) is a severe neurodegenerative disorder with early onset and a fatal outcome in young adults [1,2]. The gait instability in infancy is soon followed by a wide deterioration of the peripheral nervous system, affecting both the sensory and motor tracts. Indeed, patients experience diminution of deep tendon reflexes often leading to areflexia and a loss of the deep and superficial sensitivity. The motor deficits encompass amyotrophy, muscle weakness and evolve with skeletal deformations and loss of ambulation by the adolescence. The disease progresses with the deterioration of the central nervous system, causing ataxia, speech defect and mental retardation [3]. Defined as an axonal neuropathy by electrophysiological examination, GAN induces an axonal loss both in peripheral nerves and in the brain that is associated with the presence of enlarged axons, also called giant axons [1,2,4,5]. Strikingly, enlarged axons are filled with abnormally packed neurofilaments (NFs) that lack the proper orientation along the axon, are increased in diameter and show decreased spacing [4,6]. Whereas NFs are altered in many neurodegenerative disorders including Amyotrophic Lateral Sclerosis (ALS), Parkinson's, Alzheimer's and Charcot-Marie-Tooth diseases [7], the generalized aggregation of other neuronal and non neuronal Intermediate Filaments (IFs) [8,9] in GAN points to a central role of the *GAN *gene in maintaining cytoskeletal architecture. In particular, we used the primary fibroblasts derived from skin biopsies of multiple patients to show that vimentin aggregation is greatly enhanced in conditions driving quiescence and is not caused by an abnormal accumulation of the tubulin chaperone TBCB and its effect on microtubule stability [10,11]. After the identification of the *GAN *gene, encoding for a new BTB-Kelch protein we named gigaxonin [12], we and others mapped a total of 47 distinct mutations along the entire *GAN *gene in patients [12-23]. By revealing a high instability of gigaxonin in multiple lymphoblasts cell lines from unrelated patients [11], we confirmed that GAN is caused by a loss of function of gigaxonin.

The broad impairment of the nervous system and the generalized disorganization of IFs in GAN emphasize the crucial role of gigaxonin in maintaining neuronal and IF integrity. Moreover, gigaxonin was shown to be a direct key player in the Ubiquitin Proteasome System (UPS), as parkin in Autosomal Recessive Juvenile Parkinsonism (AR-JP) [24]. Indeed, BTB-containing proteins, including gigaxonin have been found to be the substrate adaptors of Cul3-dependant E3 ubiquitin ligases, interacting with Cul3 and the substrates through the BTB and the C-terminal domains, respectively [25-27]. The UPS is impaired in more common disorders such as ALS, Alzheimer's, Parkinson's and Huntington's diseases, but the study of its contribution to the pathology is complicated by its indirect role. Thus gigaxonin provides us with an opportunity to address directly the role of the UPS in neurodegeneration. Gigaxonin was shown to regulate *in vitro *the abundance of its three known substrates, the Microtubule (MT)-Associated Proteins MAP1B, MAP1S and the tubulin chaperone TBCB, through ubiquitin-mediated degradation by the proteasome [28-30]. Nevertheless, the modest implication of these substrates in the survival of GAN^-/- ^cortical neurons [28,29] and vimentin aggregation in patients fibroblasts [11] suggests that the mechanisms by which the gigaxonin-associated E3 ligase promotes neuronal survival and IF integrity remain to be elucidated.

In order to study the role of gigaxonin in these processes, and to evaluate the efficacy of GAN treatments that are currently under development, a relevant GAN animal model is essential. We and others independently disrupted the murine *GAN *gene in exons 3-5 (Δex3-5) [29] or promotor-exon1 (Δex1) [31] to generate gigaxonin-null mice. Whereas both published studies agree on the alteration of IFs in the nervous system of GAN^-/- ^mice, there is an important controversy concerning the degree of severity of the disease. Indeed, the GAN^Δex1 ^mouse did not develop overt neurological deficits over 15 months, suggesting a milder form of the disease in mice [31]. On the contrary, the published GAN^Δex3-5 ^model (referred here as GAN^YY^) was reported to develop strong motor deficits as early as 6 months of age, including reduction of spontaneous movement, bizarre limb positioning and overall weakness [29]. In order to characterize more precisely the phenotype of GAN^-/- ^mice, we conducted the first behavioral analysis over a 72-week period in our own GAN^Δex3-5 ^model, generated in parallel to the other studies. Monitoring of motor and sensory functions showed a mild but persistent motor impairment from 60 weeks in the 129/SvJ genetic background, while C57BL/6 animals displayed rather a deterioration of sensory functions. We tested motor deficits in the GAN^YY ^mice in parallel, and the absence of clinical signs within the first year in our hands is in agreement with a mild progression of the disease in mice, indicating that the three existing models probably display a phenotype of similar intensity. Despite the modest phenotypic manifestation and no pronounced signs of neurodegeneration, our GAN^ex3-5 ^mice exhibited severe cytoskeletal alterations. Indeed, we show here an increase in the diameter of NFs and an overt impairment in their orientation, consistent with the human disease. In addition, we found that this alteration in NF spatial organization is accompanied by a strikingly increased abundance of the three NF subunits. We also show here that gigaxonin is preferentially expressed in neuronal tissues and during development. Altogether, our results show that the absence of gigaxonin results in a milder version of the GAN disease in mice at the behavioral level, associated with a severe disorganization of the NF network that recapitulates what is observed in patients.

## Materials and methods

### Generation of GAN-deficient mice

A probe containing the murine *GAN *exons 4-5 was used to screen a BAC/PAC library (RPCI-22, Oakland, CA,  http://bacpac.chori.org/libraries.php?disp=c). For the GAN targeting vector, 1.7 kb *Ssp*I-*Xma*I and 6.2 kb *Bsa*WI-*Hind*III genomic DNA fragments homologous to 5' and 3' fragments of the mouse *GAN *gene were cloned using PGK-neo and diphteria toxin for selection. ES cells, electroporated with the linearized vector were selected with 250 μg/ml G418. One surviving clone among 400, correctly targeted accordingly to Southern blotting, was injected into C57BL/6 blastocysts to generate chimeras. Successive backcrossing of the F1 GAN^+/- ^with commercial C57BL/6 and 129/SvJ mice generated pure C57BL/6 (98,4%) and 129/SvJ (100%) GAN mice, that were interbread to obtain GAN^+/+^, GAN^+/- ^and GAN^-/- ^littermates. Animals were treated in accordance with the European Union guide for the care and the use of animals in research (commission June 18^th ^2007, 2007/526/CE). Genotyping was performed by polymerase chain reaction using three primers: the targeted fragment (600 bp) was amplified by the 5'neo (TGC GAG GCC AGA GGC CAC TTG TGT AGC) and 3'GAN primers (ATG CCA TGG ACC ATG TAT GAA GGT), and the WT fragment (700 bp) by 5'GAN (TGC CTT CCT GTC TTC CTT ATC CAC) and 3' GAN primers.

### Behavioral assay

Behavioral analysis was performed over a 72-week-period on littermate GAN^+/+^, GAN^+/- ^and GAN^-/- ^mice (15 mice per genotype, for both C57BL/6 and 129/SvJ backgrounds). All tests were performed every 4 weeks from 24 weeks of age with the appropriate devices (Bioseb, France). For Grip strength measurement, mice were allowed to grip a horizontal bar, and pulled until they released (5 trials for fore- and hind-limbs). For Rotarod analysis, mice were first trained to stay for 2 min on a bar rotating at 9 rpm. Mice were then placed on the rotating rod automatically accelerating from 4 rpm to 40 rpm for 5 min; the latency of the time to fall was recorded in 3 successive trials. In the Von Frey test, mice were first isolated in wire-mesh floor cages for 1 h. Then, the plantar surface of the hind limbs was stimulated with a set of calibrated monofilaments (3 trials per monofilament) with increasing force until the mice withdrew the limb. The minimum intensity of mechanical stimuli was taken as the force at which the mouse withdrew the paw. For the Hot plate test, mice were placed on a metal surface maintained at 53°C and the response latency to shouting, shaking, jumping or licking the hind limbs paws was measured 3 times with a 15-min interval (cut-off time: 30 sec). For the Foot print test, hind and fronts paws were painted with non-toxic finger red and blue paints. Mice then walked in a restricted cardboard tunnel with a white-paper-covered floor, and measures were taken on a sequence of 6 consecutive steps.

### Tissue preparation and morphological analysis

Mice were perfused transcardially with 4% paraformaldehyde (PFA) buffer pH 7.4 (Antigenfix, Diapath). Tissues were post-fixed O/N in 4% PFA (4°C), kept for 2 days in PBS 30% sucrose before embedding with Optimal Cutting Temperature (OCT) and kept at -80°C. For electron microscopy, tissues were kept overnight in modified Karnovsky's fixative containing 2.5% glutaraldehyde, 2.0% paraformaldehyde, 0.1% tannic acid in 0.1 M cacodylate buffer pH 7.3, post fixed 1H in 1% osmium tetroxyde then dehydrated in ethanol and finally embedded in epon. For counting motor neurons, 17 μm sections of lumbar spinal cords were cryosectionned, mounted onto slides and immunostained with anti-VaCHT antibody (SIGMA; 1:2500). Only motor neurons with a distinct absence of staining in the nucleus were counted. Sections of sciatic and L5-roots (0,5 μm) were stained with 1% toluidine blue and axons were counted manually, and normalized with the area of the nerve determined by the ImageJ software. For cytoskeletal architecture, digital electron images of ultra thin sections (80 nm) were taken with a ZEISS EM 912 microscope. The quantification of the alteration of NFs was performed by Image J, using a plug-in designed by Dr C. Matthews. The number of MTs was counted manually.

### Western blot analyses

Immunoblotting of proteins extracted from tissues was performed as described in [11]. Tissues from the GAN^ex1 ^and the GAN^YY ^mice were generously provided by Pr Julien and Dr Yang, respectively. Antibodies: mouse anti-gigaxonin [11] (N12, 1:150), rabbit anti-MAP1B-LC and anti-MAP1S-HC (kind gift from Pr Propst, 1:500), mouse anti-TBCB (AbnovaH00001155-A01, 1:250), mouse anti-NFL (Millipore MAP1615, 1:250), mouse anti- NFH (Millipore MAB5266, 1:200), mouse anti-NFM (Millipore MAB5254, 1:500), mouse anti-ßactin (Abcam 8226, 1:1000), mouse anti-GAPDH (Ambion 4300, 1:4000). Quantifications were performed with Image J after normalization with GAPDH/actin.

## Results

### Generation of the GAN^ex3-5 ^mice

**Figure 1:**
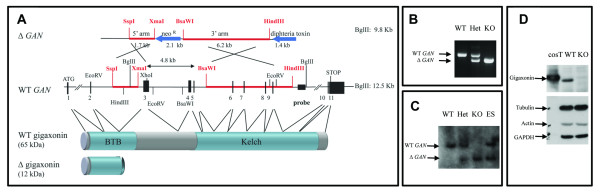
**Construction of the GAN^-/- ^mouse**. (A) Schematic representation of the disruption of the *GAN *gene. The targeted construct was designed to replace the endogenous exons 3-5 GAN locus with a neomycin-resistant gene (neo ^R^). The diphteria toxin gene, placed at the 3' end of the targeting vector allowed positive selection for the homologous recombination at the GAN locus. Proper targeting produces an open reading frame of only 110 amino acids terminated by a premature stop codon in exon 6. The Δ GAN locus will only produce a Δ gigaxonin of 12 kDa, possibly unstable and entirely lacking the Kelch domain and a portion of the BTB domain. (B, C) Analysis of the genomic DNA from Wild Type (WT), heterozygous (Het) and GAN knock out mice (KO) by polymerase chain reaction (PCR) (B) and Southern blot after BglII digestion (C). The Embryonic Stem cell (ES) corresponds to the original GAN targeted clone. (D) Brain extracts (50 μg) from WT and GAN knock out mouse, and COS cell lysates (cosT; 0,5 μg) transfected with WT-gigaxonin were analysed by immunoblot using anti-Gigaxonin antibody.

The GAN-deficient mouse was obtained by homologous recombination in the *GAN *locus in mouse ES cells. To avoid events of exon skipping within the *GAN *gene, exon 2 was not targeted because it could possibly have produced an in-frame transcript containing exons 1, 5 to 11 encoding for a full C-terminal Kelch domain. The replacement of exons 3 to 5 by a neomycin resistance gene generated a premature stop codon in exon 6, producing a truncated protein of 12 kDa, lacking a portion of the BTB domain and the entire Kelch domain (Figure 1A). The screening of ES clones performed by Southern blotting allowed the identification of homologous recombination in one single clone (Figure 1C, ES lane) that was used to generate GAN ^ex3-5 ^mice. The deletion of the *GAN *exons 3-5 was further confirmed in GAN Het and KO mice by the polymerase chain reaction and by Southern blotting (Figure 1B, C). Immunoblotting showed no detectable full-length gigaxonin in KO brain lysates, confirming the disruption of the *GAN *gene (Figure 1D).

### Preferential expression of gigaxonin during embryogenesis in neuronal tissues

**Figure 2:**
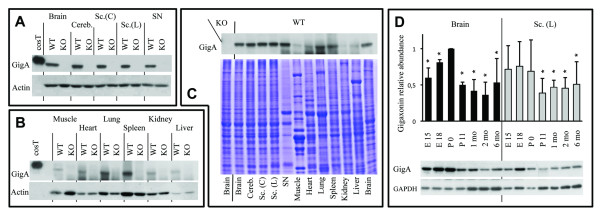
**Preferential expression of gigaxonin in neuronal tissues**. Protein extracts (50 μg) of neuronal (A) and non neuronal (B) tissues from 24 week-old WT and GAN KO mice were immunoblotted with anti-gigaxonin (GigA) and anti-Actin antibodies. Cereb. = cerebellum; Sc. (C) = cervical section of spinal cord; Sc. (L) = lumbar section of spinal cord; SN = sciatic nerve. Specificity of detection is demonstrated by the absence of immunoreactivity in the tissues from KO mice. Note that the abundance of actin (comparable between WT and KO mice) varies in non neuronal tissues. The relative abundance of gigaxonin in multiple tissues was assessed by immunoblotting with GigA antibody (C, top panel). Coomassie blue staining indicates the equal loading of total proteins in all the tissues (C, bottom panel). (D) Temporal expression of gigaxonin, from embryonic stage E15 to adult stage (6 months) was determined in brain and lumbar sections of WT mice by immunoblotting with GigA, and normalized with anti-GAPDH antibody (n = 3; Mann-Whitney test, *, p < 0.05; bars represent standard deviation).

Monoclonal antibodies, previously developed to reveal gigaxonin's low abundance in brain [11], were used to assess its spatio-temporal expression in different mouse tissues. Gigaxonin was expressed evenly throughout the nervous system but with very low levels of expression in muscle, heart, kidney and liver (Figure 2A-C). Its temporal expression, assessed in brain and lumbar section of spinal cord from embryonic stage E15 to 6 months of age, revealed that the high levels of gigaxonin detected in embryonic stages up to PO, dropped by 50-56% in both tissues after birth, and then stayed constant in adults.

### GAN^ex3-5 ^mice show mild motor and sensory impairments

Considering the massive deterioration of the sensory-motor functions in patients [1,2], leading to loss of ambulation and to the loss of the deep and superficial sensitivity, we investigated the progression of the disease in gigaxonin-depleted mice over time.

Thus, we conducted the first behavioral study on GAN mice over a 72-week period using motor and sensory tests, on a homogeneous cohort of GAN^ex3-5 ^mice in the original pure 129/SvJ background.

**Figure 3:**
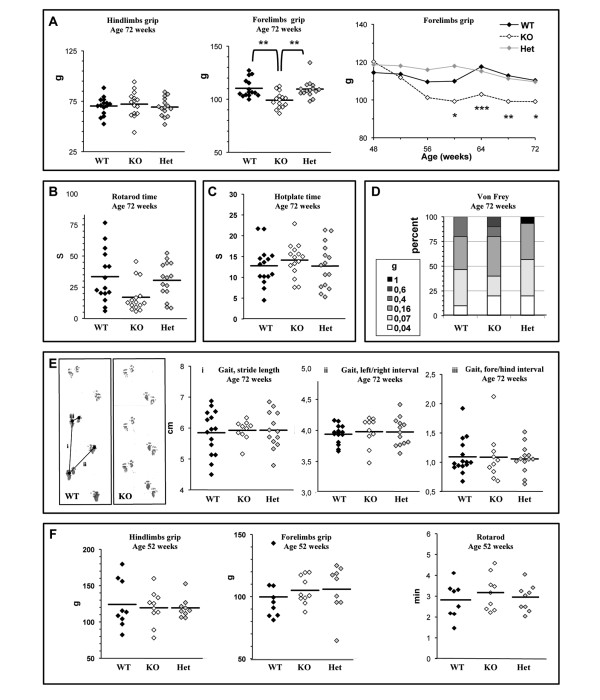
**GAN mice present persistent motor deficits**. Motor functions were evaluated with a Grip Strength test (A), and a Rotarod test (B). Sensory deficits were recorded with a Hot plate test (C) and Von Frey filaments test (D). Each test was performed every 4 weeks over a 72 week-period (n = 15 mice per genotype). The scores of individual mice are represented at 72 weeks with the mean score represented by a bar for each genotype. The average score was also represented over time for the Forelimb grip test, to show the statistical significance of motor impairment in the GAN mice from 60 weeks of age (two-way ANOVA with Bonferroni post-test: *, p < 0,5; **, p < 0,01 and ***, p < 0,001). Gait analysis of the GAN mice at 72 weeks of age (E). The stride length (i), the width between the right and left paws (ii) and the overlap between the forepaws and hindpaws was measured for each mouse (n = 15 per genotype) and averaged (bar). Analysis of the other GAN^ex3-5 ^model (GAN ^YY^) did not reveal any motor deficits within the first year of age (F) (n = 10 per genotype).

At 72 weeks of age, GAN^ex3-5 ^mice displayed a specific impairment in the Grip strength in the forelimbs, with mean values of 110 ± 8,68 and 109,58 ± 8,26, respectively, for the WT and Het mice and of 99,15 ± 7,48 for KO mice (Figure 3A). This motor deficit is mild compared to humans but persists over 4 consecutive months, starting at week 60 (Figure 3A, right panel). Analysis of the motor activity of GAN^ex3-5 ^mice by the Rotarod test showed no significant difference between WT and gig-null mice, although the last data set (week 72) showed a strong tendency towards a decline for KO mice (Figure 3B). We did not detect significant changes in the average sensitivity to temperature in GAN^ex3-5 ^mice over the 72-week period (Figure 3C), although a single KO mouse developed a strong insensitivity over the last five consecutive months. Sensitivity to mechanical stimuli was not impaired in GAN^ex3-5 ^mice, as the retraction of paws upon filaments pressure did not significantly differ between WT and KO mice during the entire study (Figure 3D).

As cerebellar functions are impaired in GAN patients, we recorded the gait of GAN^ex3-5 ^mice using a footprint test (Figure 3E). Measurements of the stride length, the width between left and right paws and the overlap between fore and hind paws at 72 weeks of age did not reveal any differences between the WT, Het and KO mice that performed the test. However, 34% of the KO mice and 14% of the Het mice that were otherwise moving normally could not be evaluated due to abnormal pausing or refractory behavior to enter in the tunnel. Finally, neither animal weight nor viability was affected by gigaxonin depletion over the 72-week period (data not shown).

To assess for a possible phenotypical variability in different genetic backgrounds, we performed a similar behavioral study in GAN^ex3-5 ^mice derived in a 98,4% pure C57BL/6 background at 24, 48 and 72 weeks of age. Interestingly, we revealed a mild sensory deficit in the GAN^ex3-5 ^mice in this line, but no effect on motor abilities. Indeed, C57BL/6 GAN^ex3-5 ^mice were significantly less sensitive to heat, with a mean time spent on the hot plate of 10,40 ± 5,41; 9,43 ± 5,52 for the WT mice and 15,95 ± 5,66; 15,41 ± 5,92 for the KO mice at 48 and 60 weeks of age, respectively (Additional file 1). Three of the KO mice developed the highest insensitivity from 48 weeks of age, with individual times repeatedly approaching the time cut limit. Gait, animal weight and survival were not compromised in C57BL/6 GAN^ex3-5 ^mice.

Another GAN model (GAN^YY^) with deletion of exons 3 to 5 in the *GAN *gene was reported to develop a progressive and severe deterioration of motor functions from 6 months of age [29]. In this study, GAN^YY ^mice displayed a reduction of spontaneous movements, bizarre limb positioning and overall weakness, followed by muscle wasting and weight loss. To assess for a possible phenotypic difference between the two GAN^ex3-5 ^models, we performed the same motor behavioral tests used for our model on GAN^YY ^mice. In our hands, GAN^YY ^mice do not show any motor deficits over a 52 week-period, as revealed by normal grip strength measurements and unaltered motor activity on the Rotarod test (Figure 3F). Difficulty in walking, fragile hair or absence of whiskers, previously reported in the GAN^YY ^model [29], were occasionally observed in control mice and at a similar and normal frequency in both GAN models (GAN^YY ^and GAN^ex3-5^) (data not shown). Lastly, in our hands GAN^YY ^mice did not display any abnormal weight loss compared to the controls over the course of the experiment (data not show).

### Preservation of lumbar motor neurons and axons in GAN^ex3-5 ^mice

**Figure 4:**
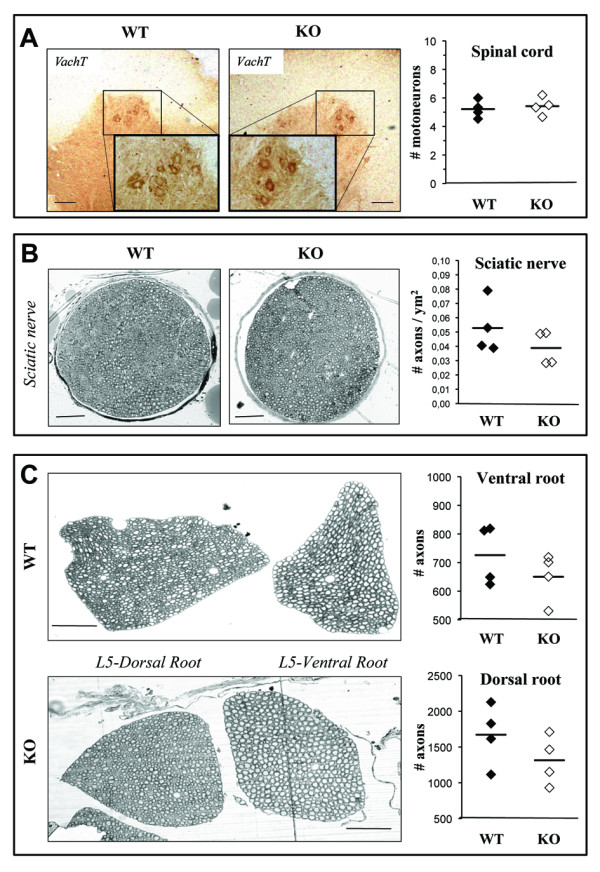
**Lumbar motor neurons and axons are preserved in GAN mice**. (A) Cross sections of lumbar spinal cord from 48-week-old WT and GAN mice, stained with VaCht (n = 4 animals). Inserts show lower magnification. (B-C) Cross sections of sciatic nerve (B), L5 dorsal and ventral roots (C) from 48-week-old WT and GAN mice, stained with toluidine blue. The numbers of distal axons (B), proximal motor and sensory axons (C) are represented for each mouse (n = 4 per genotype) and averaged (bar). Bars = 50 μm (A, B, C).

To assess whether gigaxonin ablation produces, as in humans, a neuronal loss in the ventral horn of the spinal cord [32], a decrease of axonal density in peripheral nerves and a presence of giant axons [4,5], we examined cross sections of spinal cords and proximal/distal nerves from 48 week-old GAN^ex3-5 ^mice. Regardless of the genetic background, the number of motor neurons in lumbar spinal cord did not differ between WT and KO mice (Figure 4A and Additional file 2). Similar experiment performed at 72 weeks did not reveal any significant motor neurons loss in 129/SvJ GAN^ex3-5 ^mice (data not shown). The counting of axonal content, distally in the sciatic nerve and proximally in the ventral and dorsal roots of lumbar sections L5 showed a constant tendency towards a decrease in axonal density in the original 129/SvJ background but not in the C57BL/6 background (Figure 4B, C and Additional file 2). No giant axons were observed in any of these tissues.

### Severe alteration of cytoskeletal architecture in GAN^ex3-5 ^mice

**Figure 5:**
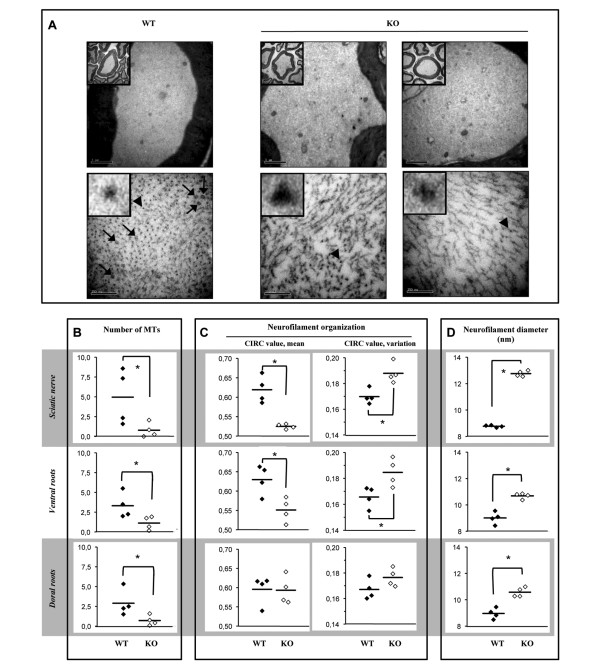
**Severe disorganization of cytoskeletal architecture in GAN mice**. (A) Electron microscopic examination of the axoplasm of GAN nerves revealed a diminution in microtubule content (arrows), an abnormal orientation and an increase in the diameter of neurofilaments (individual neurofilaments indicated by an arrowhead are magnified in the inserts). (B-D) The quantification of the cytoskeletal alteration in 48 week-old GAN mice was performed in sciatic nerves, L5-ventral and dorsal roots (n = 4 mice per genotype; 4 axons per mouse; 3 random pictures of distinct regions per axon; representing a total of 12 fields per mouse). (B) The mean number of microtubules per field was significantly lower in GAN compared to WT nerves (*, p < 0.05, Mann-Whitney test). (C) The alteration of neurofilament orientation was assessed by the measurement of the circularity of individual neurofilaments (circ = 1 and circ<1 representing a perfect circle and an elongated shape, respectively). The left panel displays average circularity scores, measuring the general orientation of the neurofilaments, for individual mice in the three tissues (the mean score per genotype is represented by a bar). The right panels show the standard deviations of the average circularity scores for the same analysis, a measure that is representative of the variations in the orientation of individual neurofilaments within each tissue section (*, p < 0.05, Mann-Whitney test). (D) Neurofilament diameter is significantly increased in GAN mice (10 individual neurofilaments per field; *, p < 0.05, Mann-Whitney test).

In human, GAN induces a profound alteration of IF architecture. Inside the nervous system, the organization of NFs is severely impaired: they exhibit an abnormal compaction, an alteration in longitudinal orientation and an increase in diameter [4,6]. To evaluate the effect of gigaxonin depletion on cytoskeletal architecture in mice, we conducted an ultrastructural analysis of proximal and distal nerves in our GAN^ex3-5 ^mouse model. This revealed a profound impairment in cytoskeletal organization in GAN^ex3-5 ^mice at 48 weeks of age, and in both 129/SvJ and C57BL/6 backgrounds (Figure 5 and Additional file 3).

In the 129/SvJ mice, the axoplasms of GAN^ex3-5 ^present a 6,25-, 2,9- and 4-fold decrease in the number of microtubules in sciatic nerves, L5-ventral and dorsal roots, respectively (Figure 5A, B). In the absence of gigaxonin, NFs are abnormally distributed and lose their regular and parallel orientation along the axons, displaying either mixed populations of well-oriented and misoriented NFs or completely disorganized NFs (Figure 5A). This alteration in NF orientation was quantified by measuring the circularity (CIRC) of the sections of individual NFs on micrographs. The mean CIRC values, for which the highest possible value of 1 represents an orientation of all NFs perfectly parallel to the axon, were found to be significantly lower in gigaxonin-null tissues than in controls (sciatic: WT = 0,62 ± 0,003; KO^- ^= 0,52 ± 0,006, L5-ventral root: WT = 0,62 ± 0,003; KO^- ^= 0,55 ± 0,03. Figure 5C, left panels). Furthermore, the standard deviation of the CIRC scores within each image, a measure that is indicative of differences of orientation of individual NFs within a given axon, was significantly higher in the GAN mice than in the controls (Figure 5C, right panels). In addition, we showed that the diameter of the properly orientated NFs significantly increased in GAN^ex3-5 ^axoplasms, from 8,77 ± 0,08 nm for the controls to 12,76 ± 0,22 nm; 9,00 ± 0,46 nm to 10,68 ± 0,21 nm; 8,96 ± 0,40 nm to 10,57 ± 0,36 nm in GAN^ex3-5 ^sciatic, L5-ventral and dorsal roots, respectively (Figure 5D).

We revealed a similar alteration of MT content, NF orientation and diameter in C57BL/6 GAN^ex3-5 ^mice (Additional file 3).

### Profound alteration of NF protein levels in GAN models

**Figure 6:**
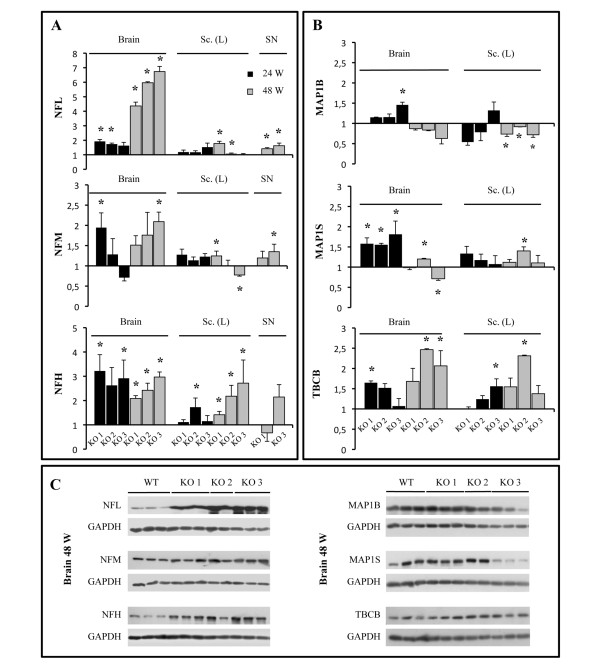
**Increased abundance of NF subunits in the three GAN models**. The relative increase of protein content was obtained by comparing the mean abundance in each of the three GAN models (KO1 = our GAN^ex3-5^; KO2 = GAN^YY^; KO3 = GAN^ex1^) with WT mice (n = 3 mice per genotype, except n = 2 for 48 week-old KO2). (A) Expression levels were quantified in the brain, the lumbar section of spinal cord (Sc-L) and sciatic nerves (SN) by immunoblotting using anti-NFL, NFM and NFH antibodies and normalization with GAPDH antibody. (B) The relative abundance of the gigaxonin's partners MAP1B, MAP1S and TBCB was quantified using the corresponding antibodies, with a similar approach. (Mann-Whitney test, *, p < 0.05; bars represent standard deviation). The immunoblots corresponding to the abundance of the NF subunits and the gigaxonin's partners in brain of 48 week-old GAN models are represented in (C).

To assess whether the impaired distribution of NFs is associated with an increased abundance of NF proteins, we quantified the expression level of the three subunits NF-L, NF-M and NF-H in mouse brain, spinal cord and sciatic nerves, in the three gigaxonin-null mice: (our GAN^ex3-5 ^mouse, the GAN^YY ^and the GAN^ex1 ^mice). The analysis revealed an increased abundance of all three NF subunits, in all tissues and at all ages (Figure 6A, C). For all three NF subunits the maximum increase, ranging from 2-3 fold, was detected in brain tissues, with 1,5-2,5 fold increases in spinal cord and sciatic nerves. Interestingly, whereas the overabundance of NF-M and NF-H is constant overall from 24 to 48 weeks of age, a spectacular increase of NF-L is observed at 48 weeks of age specifically in the brain (Figure 6A, C). Indeed, the abundance of NF-L is increased 4,3- to 6,7- fold in the brain of the three GAN models.

Finally, we determined the expression levels of gigaxonin's known partners MAP1B, MAP1S and TBCB, that were shown to be increased 1.5- to 3-fold for MAP1B/MAP1S and much higher for TBCB in GAN^YY ^mice [28-30]. Our analysis confirmed a less than 2-fold increase of MAP1S in GAN brain, while in our hands the level of TBCB was only increased by less than 3-fold compared to controls, and MAP1B abundance was not significantly affected, in all three murine GAN models and at both ages tested (Figure 6B, C).

## Discussion

This report of the behavioral analysis on aging mice disrupted in the *GAN *gene evaluates for the first time the deterioration of motor and sensory functions, both of which are impaired in human GAN. Our analysis reveals a late onset but robust motor impairment over time, affecting preferentially the grip strength in the forelimbs from 60 weeks of age in a pure 129/SvJ background. The fact that a single mouse showed a persistent decrease in sensitivity to thermal stimulus might also suggest a possible very low penetrance of sensory dysfunction. Sensory deficits developed over time in the C57BL/6 GAN animals that in turn displayed no motor deficits, which may indicate a modulation of the phenotype by the genetic background. Our results are in agreement with the absence of weakness in the hind limbs monitored over 15 months in another GAN model (GAN^ex1^) [31]. In our hands, no motor deficits were observed in the first year of life in the third GAN model (GAN^YY^) [29], previously reported to develop a progressive and strong deterioration of motor functions from 6 months of age. Overall, it is very likely that all three models develop a similar, mild form of the disease, with a late (≥ 1 year) onset and a slow progression that does not totally abolish ambulation and sensitivity as in humans.

Whereas the rare autopsies of GAN patients have revealed a loss of neurons in the ventral horn of the spinal cord [32], no motor neuron degeneration was observed in our GAN^ex3-5 ^model, in agreement with observations in the GAN^ex1 ^model at 48 weeks of age [31]. The decrease in the axonal density in proximal and distal nerves of our GAN^ex3-5 ^129/SvJ mice was constant but approached statistical significance and would have required an increase in animal numbers. Nevertheless, this observation is in accordance with the 27% axonal loss observed in ventral roots of the GAN^ex1 ^model [31]. We did not observe enlarged axons, in agreement with both previously described models [29,31]. Thus, it appears that gigaxonin-null mice do not mimic the axonal defects that are hallmarks of human GAN, at least at the ages that were studied. Overall, it is probable that GAN is not fully expressed in mouse because of genic compensation (by another E3 ligase or substrate adaptor), the short life span of the animals, the smaller length of the axons or indeed environmental factors.

Regardless of the genetic background, we showed that absence of gigaxonin in mice has a striking impact on cytoskeletal architecture. Extending initial observations [30], we reveal a significant decrease in MT content (up to 84%) both in proximal and distal GAN nerves. It has been proposed that the reduction of MT density could solely result from the accumulation of the gigaxonin's substrate TBCB [30]. However, by quantifying its effect in cell lines and human primary fibroblasts, we already have shown that even in an overexpression system, TBCB is not a potent destabilizing agent of MTs [11]. Moreover, we show here, that its abundance in the three GAN models at different ages increases only modestly in older mice, in contrary to the massive increase that was previously reported [30]. Thus, we suggest that TBCB accumulation might play a part in the diminution of MT density in GAN, but that other factors such as an alteration of the NF network are likely to be relevant.

Indeed, the most striking phenotype observed in our GAN mouse model in both genetic backgrounds is the broad and massive alteration of NF organization that extends to proximal and distal nerves. Whereas NFs were reported to be packed in the GAN^YY ^model [29], we show here for the first time the spatial disorganization and increased diameter of NFs, two features that are seen in patients [4]. By evaluating the orientation of NFs by a measure of the circularity on transversal NF sections, we show that gigaxonin-null NFs loose their parallel orientation along the axon. Additionally, the increase of the diameter of NFs from 9 nm in control animals to 10-12 nm in our mice is consistent with the enlargement from 10 to 12 nm seen in patients [4]. These alterations of NF architecture come with a broad increase of the abundance of all NF subunits, as reported previously in brain, cerebellum and spinal cord tissues of GAN^ex1 ^mice [31]. We show here that the alteration in NF content is comparable in the three GAN models, is more pronounced in the brain for all subunits, and reaches a spectacular 4- to 7-fold increase for NFL in 48 week-aged mice. The slight increase of NFL in sciatic nerves is comparable to the 2-fold increase that was revealed in the sural nerve of a patient [33]. Nevertheless, the absence of obvious compaction of NFs in our GAN mouse, in contrary to the 2-fold decrease in NF spacing in patients [4,6], suggests either a milder disorganization in rodents or reflects differences in NF architecture between humans and mice. Interestingly, the cytoskeletal alterations occur prior to the neuronal dysfunction (as shown by the phenotypic deficits), allowing speculation that NF disorganization may have a causative role in the development of the mild motor and sensory deficits in our GAN mouse.

Thus, the full expression of the disease in GAN mice, required to perform preclinical therapeutic studies, might be achieved by exacerbation of NF collapse, by genetically modulating NF stochiometry or by using chemical neurotoxins [7,34] such as acrylamide, that produces a neuropathy resembling human GAN with accumulation of NFs [35].

## Conclusions

Our results show that gigaxonin depletion in mice causes a mild form of the GAN disease, characterized by a late onset and a slow progression of sensory and motor symptoms. Nevertheless, the striking alteration of neurofilament architecture in gigaxonin-null mice closely resembles the human pathology. Indeed, the increased abundance of neurofilaments is associated with a spatial disorganization and an increase in diameter. Thus, our mouse model will be useful to decipher the pathological mechanisms by which the gigaxonin-E3 ligase maintains neuronal survival and cytoskeletal integrity. Elucidating the yet unknown pathological mechanisms at stake in GAN is not only imperative for the patients, but it may also advance our understanding of how defects of the Ubiquitin Proteasome System and neurofilament network, impaired in more common disorders such as ALS, Huntington's, Alzheimer's and Parkinson's diseases, participate in neurodegeneration.

## Competing interests

The authors declare that they have no competing interests.

## Authors' contributions

TG carried out the behavioral and histopathological analysis and in part the electron microscopy study; AB carried out the immunoblotting; RB participated in the analysis of the electron microscopy study, performed statistical analysis and helped to draft the figures and the manuscript; JPC prepared the samples and provided assistance for the electron microscopy study; PB constructed the GAN mouse, conceived and coordinated the project, participated in the analysis of the behavioral and electron microscopy studies, drafted the figures and the manuscript. All authors read and approved the final manuscript.

## Supplementary Material

Additional file 1 **C57BL/6 GAN mice present sensory deficits**. Motor functions were evaluated with a Grip Strength test (A), and a Rotarod test (B). Sensory deficits were recorded with a Von Frey test (C) and Hot plate test (D). Gait analysis was assessed by a Footprint test (E). Each of the sensory/motor tests was performed at 24, 48 and 72 weeks of age, with additional analysis at 60 weeks of age for the Hot Plate test (n = 15 mice per genotype). The average scores, represented over time for the Hot plate test show the statistical significance of sensory impairment in the GAN mice from 48 weeks of age (two-way ANOVA with Bonferroni post-test: *, p < 0,5). Click here for file

Additional file 2 **Lumbar motor neurons and axons are preserved in C57BL/6 GAN mice**. As described for the 129/SvJ line (see Figure 4), the number of motor neurons (A) and distal (B) and proximal axons (C) was determined in lumbar spinal cord, sciatic and L5 dorsal/ventral roots sections from 48-week-old WT and GAN mice, respectively. (n = 4 per genotype). Bars = 50 μm (A, B, C). Click here for file

Additional file 3 **Severe disorganization of cytoskeletal architecture in C57BL/6 GAN mice**. (A) Electron microscopic examination of the axoplasm of GAN nerves revealed a diminution of MTs (arrows), an abnormal orientation and an increase in the diameter of neurofilaments (Individual Neurofilaments indicated by an arrowhead are magnified in the inserts). (B-D) The quantification of the cytoskeletal alterations in 48 week-old GAN mice was performed as described for the 129/SvJ GAN line (see Figure 5) in sciatic nerves, L5-ventral and dorsal roots. (*, p < 0.05, Mann-Whitney test). Click here for file
