# Supplementary figures and images for: Sensory-motor deficits and neurofilament disorganization in gigaxonin-null mice
Source: Mol Neurodegener. 2011 Apr 12;6:25. doi: 10.1186/1750-1326-6-25 (PMC3094382; doi:10.1186/1750-1326-6-25)

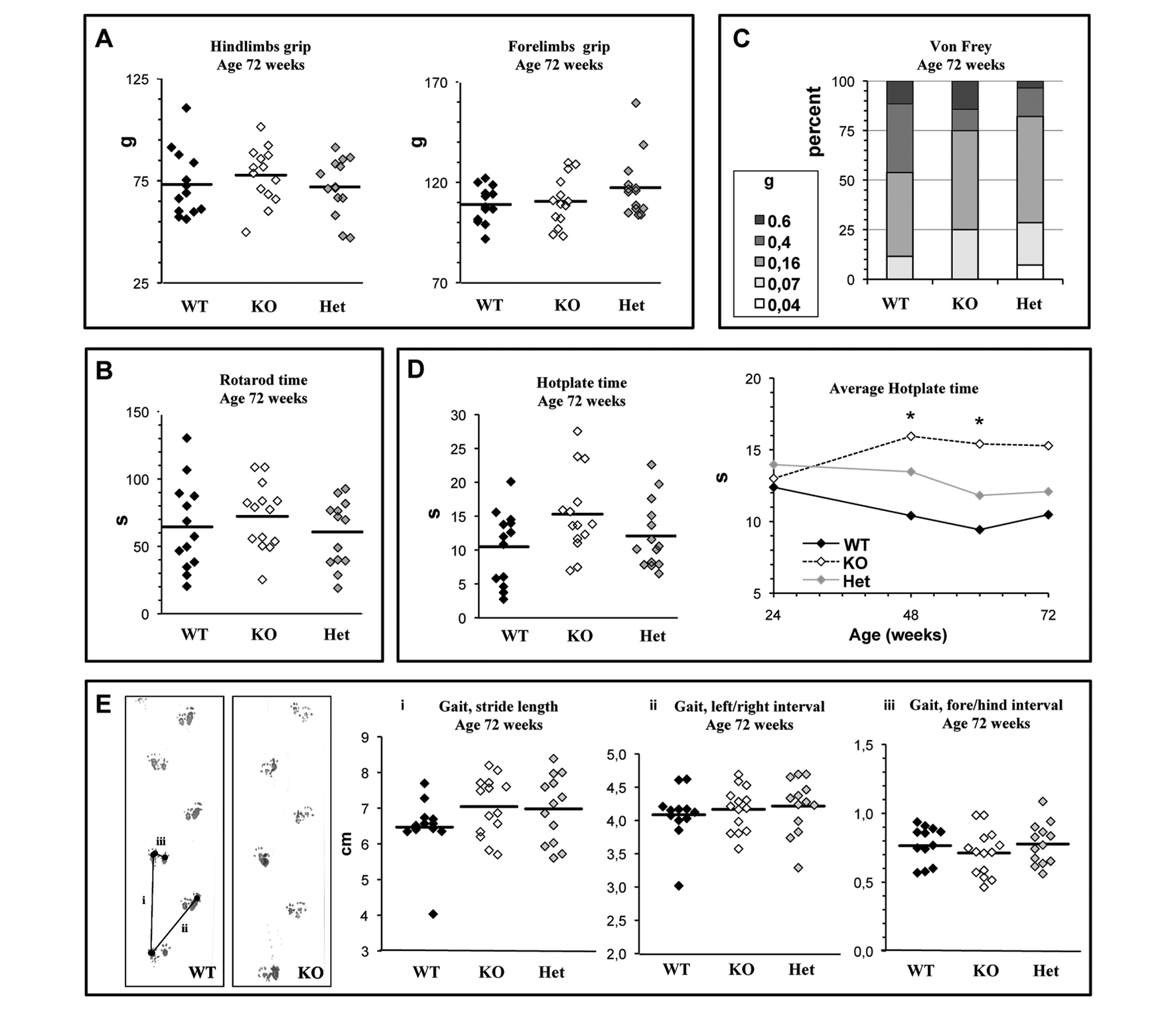

Supplement: Additional file 1 — C57BL/6 GAN mice present sensory deficits. Motor functions were evaluated with a Grip Strength test (A), and a Rotarod test (B). Sensory deficits were recorded with a Von Frey test (C) and Hot plate test (D). Gait analysis was assessed by a Footprint test (E). Each of the sensory/motor tests was performed at 24, 48 and 72 weeks of age, with additional analysis at 60 weeks of age for the Hot Plate test (n = 15 mice per genotype). The average scores, represented over time for the Hot plate test show the statistical significance of sensory impairment in the GAN mice from 48 weeks of age (two-way ANOVA with Bonferroni post-test: *, p < 0,5). [file 1750-1326-6-25-S1.TIFF]

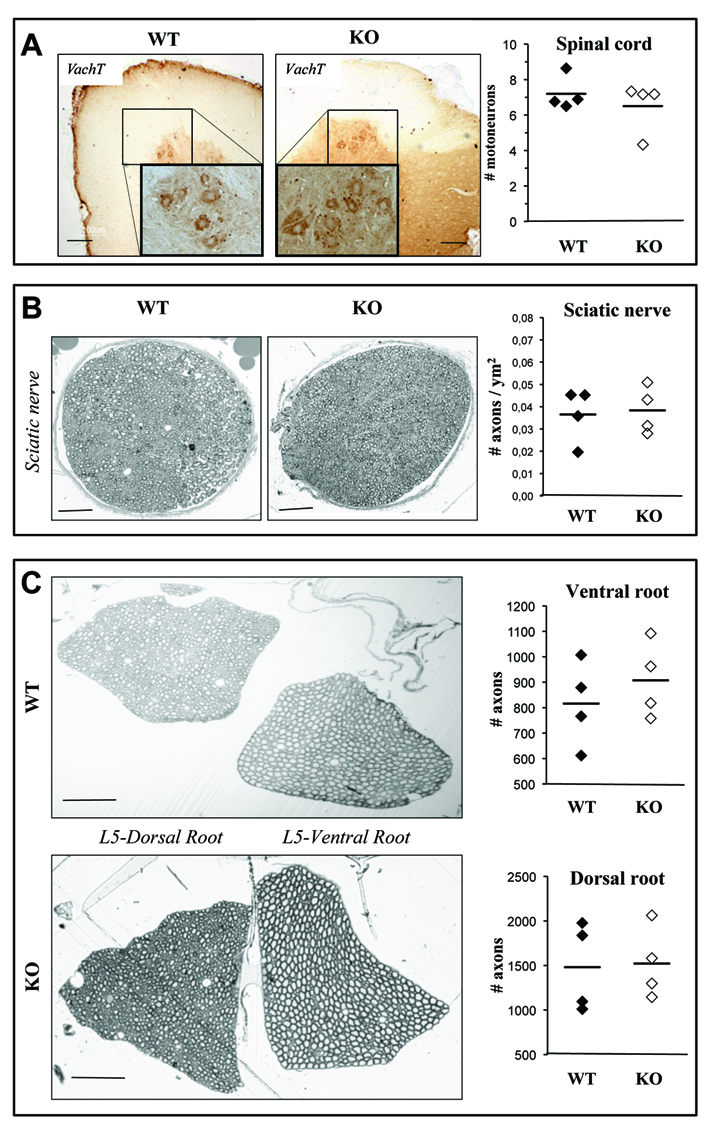

Supplement: Additional file 2 — Lumbar motor neurons and axons are preserved in C57BL/6 GAN mice. As described for the 129/SvJ line (see Figure 4), the number of motor neurons (A) and distal (B) and proximal axons (C) was determined in lumbar spinal cord, sciatic and L5 dorsal/ventral roots sections from 48-week-old WT and GAN mice, respectively. (n = 4 per genotype). Bars = 50 μm (A, B, C). [file 1750-1326-6-25-S2.TIFF]

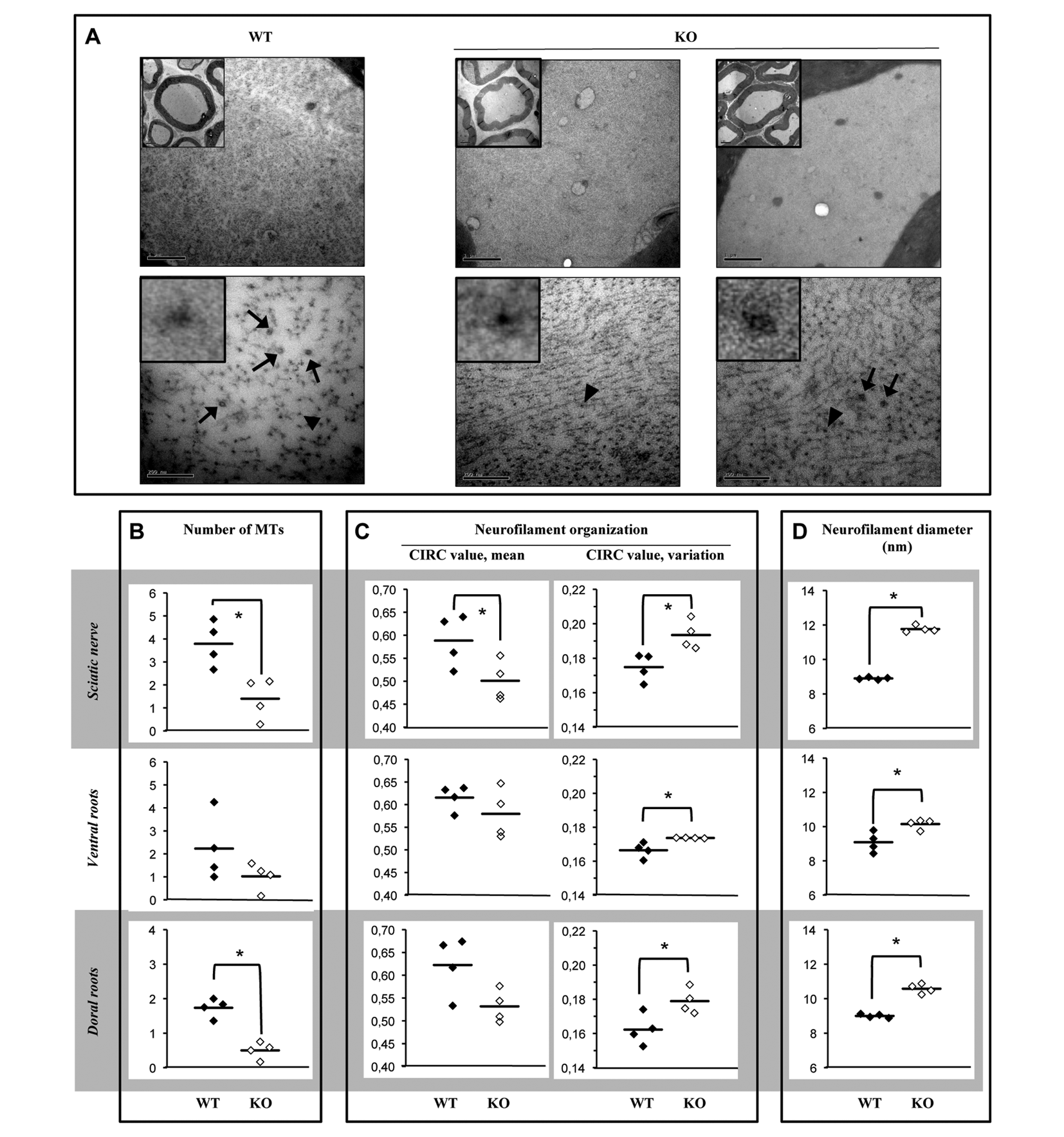

Supplement: Additional file 3 — Severe disorganization of cytoskeletal architecture in C57BL/6 GAN mice. (A) Electron microscopic examination of the axoplasm of GAN nerves revealed a diminution of MTs (arrows), an abnormal orientation and an increase in the diameter of neurofilaments (Individual Neurofilaments indicated by an arrowhead are magnified in the inserts). (B-D) The quantification of the cytoskeletal alterations in 48 week-old GAN mice was performed as described for the 129/SvJ GAN line (see Figure 5) in sciatic nerves, L5-ventral and dorsal roots. (*, p < 0.05, Mann-Whitney test). [file 1750-1326-6-25-S3.TIFF]
